# Transverse Coherence Limited Coherent Diffraction Imaging using a Molybdenum Soft X-ray Laser Pumped at Moderate Pump Energies

**DOI:** 10.1038/s41598-017-05789-w

**Published:** 2017-07-13

**Authors:** M. Zürch, R. Jung, C. Späth, J. Tümmler, A. Guggenmos, D. Attwood, U. Kleineberg, H. Stiel, C. Spielmann

**Affiliations:** 1 Institute of Optics and Quantum Electronics, Abbe Center of Photonics, Friedrich Schiller University Jena, Max-Wien-Platz 1, 07743 Jena, Germany; 2 University of California Berkeley, Chemistry Department, Berkeley, CA 94720 USA; 3 Helmholtz Institute Jena, Fröbelstieg 3, 07743 Jena, Germany; 4 Max-Born Institute, Max-Born Str. 2A, D-12489 Berlin, Germany; 5 Ludwig-Maximilians-Universität München, Am Coulombwall 1, D-85748 Garching, Germany; 6 Max-Planck-Institut für Quantenoptik, Hans-Kopfermann-Str. 1, D-85748 Garching, Germany; 7 University of California Berkeley, Department of Electrical Engineering and Computer Sciences, Berkeley, CA 94720 USA

## Abstract

Coherent diffraction imaging (CDI) in the extreme ultraviolet has become an important tool for nanoscale investigations. Laser-driven high harmonic generation (HHG) sources allow for lab scale applications such as cancer cell classification and phase-resolved surface studies. HHG sources exhibit excellent coherence but limited photon flux due poor conversion efficiency. In contrast, table-top soft X-ray lasers (SXRL) feature excellent temporal coherence and extraordinary high flux at limited transverse coherence. Here, the performance of a SXRL pumped at moderate pump energies is evaluated for CDI and compared to a HHG source. For CDI, a lower bound for the required mutual coherence factor of |*μ*_12_| ≥ 0.75 is found by comparing a reconstruction with fixed support to a conventional characterization using double slits. A comparison of the captured diffraction signals suggests that SXRLs have the potential for imaging micron scale objects with sub-20 nm resolution in orders of magnitude shorter integration time compared to a conventional HHG source. Here, the low transverse coherence diameter limits the resolution to approximately 180 nm. The extraordinary high photon flux per laser shot, scalability towards higher repetition rate and capability of seeding with a high harmonic source opens a route for higher performance nanoscale imaging systems based on SXRLs.

## Introduction

Short wavelength laser sources in the extreme ultraviolet (XUV) and soft X-ray (SXR) regime have validated their relevance for microscopy applications in fundamental and life sciences in the past decade. Serial crystallography at free electron lasers^1^ has become an important technique for the determination of protein structures^2^ but also enabled insights into biologic objects such as viruses^3^. Table-top XUV sources employing high-order harmonic generation (HHG)^4^ have proven their relevance to the field by allowing a broader access to short wavelength imaging and holography^5^ and the implementation of more complex experimental schemes. The reflection geometry^6^ for instance has become a technique for surface structure analysis^7, 8^ and biologic specimen classification^9^ and promises many new applications in the near future. A major drawback of HHG sources often addressed is the limited flux demanding for integration time of several seconds up to hundreds of seconds^10–15^ depending on the anticipated resolution of the XUV microscope. On the other hand, the transverse coherence of HHG sources is excellent, whereas the temporal coherence is determined by the duration of the driving laser pulse as well as the bandwidth of the applied XUV optics translating into the bandwidth of the applicable high harmonic radiation^16^. For sufficiently narrow-band HHG radiation a spatial resolution below the wavelength of illumination near the Abbe limit can be achieved^17, 18^.

In turn, plasma-based soft X-ray lasers (SXRL) emitting short pulses in the XUV range between 3 and 40 nm (ref. 19) are table-top sources with high single-shot photon flux. Among the numerous schemes proposed for soft X-ray lasing, the transient collisional excitation scheme has proved to be the most reliable and promising for the development of compact laser-pumped SXRLs^20^. Combining this scheme with the so-called grazing incidence pumping (GRIP) geometry compact systems are feasible^21, 22^. Using the GRIP scheme strong XUV emission in the range between 10 and 20 nm with pulse energies of the SXRL up to 3 µJ have been demonstrated for pump laser energies of about 1.5 J (refs 23 and 24). Due to the properties of the gain medium the spectral bandwidth of the XUV emission is in the order of a few picometer resulting in a very high temporal coherence^25^. The transverse coherence of the SXRL strongly depends on the geometry of the excitation scheme, i.e. one versus two stage amplifiers or seeded versus unseeded operation, and the pumping conditions. In dependence on the pumping conditions the highly coherent part of the beam contains between 1% (unseeded, pump energy 1–2 J, ref. 26) and 90% (seeded or two stage SXRL with pump energies up to 10 J, refs 27 and 28) of the total SXRL energy. There are only very few data sets concerning the transverse coherence of a single stage, and thus more easily realizable, SXRL pumped at moderate pump energies below 1 J (ref. 29). On the other hand, these systems are relatively attractive for applications requiring high average photon flux, since pump lasers with pulse energies below 1 J are easily scalable to repetition rates up to 200 Hz.

There are only very few examples for coherent diffraction imaging (CDI) experiments using laser plasma-based SXRL in literature. Kang *et al*. demonstrated^30^ a SXRL operating at 13.9 nm pumped by a 1.5 J, 10 Hz Ti:Sapphire laser and its application to coherent diffraction imaging. They achieved a resolution in the order of 100 nm.

Here, we investigate the applicability of SXRLs towards CDI in the light of the high XUV pulse energy, the excellent temporal coherence and the limited transverse coherence. While there is a wide array of previous publications discussing CDI with partially coherent sources^31, 32^ in-depth and modifications to established reconstruction algorithms^33, 34^, we limit ourselves in this work to standard plane wave CDI methods in order to retrieve the optical properties of the SXRL and subsequent limitations towards applications such as CDI. We demonstrate that the captured diffraction data of a known object allows estimating the transverse coherence, which is compared to a quantitative measurement of the transverse coherence employing double slits. Moreover, we directly compare the SXRL-CDI measurement to a HHG-CDI measurement within the same instrument to benchmark the performance. Finally, this paper outlines a concept and operation parameters for a SXRL pumped at moderate energies.

## Experimental setup and properties of the SXRL

**Figure 1:**
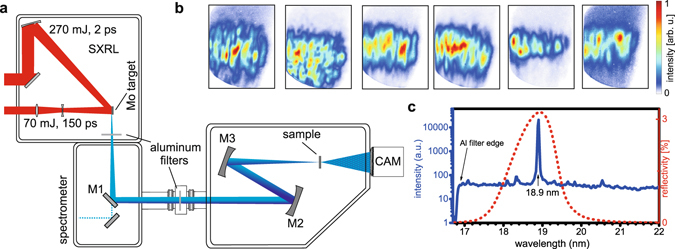
Experimental layout and SXRL properties. (**a**) The SXRL radiation is generated using a molybdenum target. The plasma is created by a long pre-pulse (70 mJ, 150 ps). The main pulse (270 mJ, 2 ps) prepares the population inversion in the highly excited nickel-like ions. Aluminium filters (500 nm thickness in total) suppress infrared stray light. Two curved mirrors (M2 & M3) reimage the light onto the sample and demagnify it by a factor of two. A soft X-ray sensitive CCD (CAM, ANDOR model iKon L) captures the far-field diffraction pattern of the sample. (**b**) Typical far-field modes comprising of single SXRL shots measured with the sample removed. (**c**) Typical Mo SXRL spectrum peaked at 18.9 nm (blue line) and combined reflectivity of the multilayer mirrors (M1, M2 and M3, red dotted line).

The experimental setup is depicted in Fig. 1a. The SXRL consists of a molybdenum target that is pumped with a 70 mJ/150 ps long pre-pulse and 270 mJ/2 ps short pulse, respectively, at a repetition rate of 100 Hz (see Methods for further details). Typical far-field mode profiles feature a rectangular shaped beam profile, with higher frequency modulations hinting at the plasma and single pass nature of the source (Fig. 1b). The generated soft X-ray radiation passes a plane molybdenum-silicon multilayer mirror (M1, reflectivity approximately 22%, bandwidth 2.5 nm) at 45 degree and is refocused onto the sample for coherent diffraction imaging by two concave spherical mirrors (radius of curvature (ROC), ROC_2_ = 2 m, ROC_3_ = 1 m) with a molybdenum-silicon multilayer coating near normal incidence. The design of all mirrors was chosen such that the reflectivity peaks at the central wavelength according to the SXRL emission line at 18.9 nm. The mirrors were additionally optimized for reflectivity for higher throughput, while the total bandwidth is mainly determined by the strong monochromatic SXRL molybdenum emission line^35^. The curved multilayer mirrors (M2 and M3) have been realized with a dual ion beam deposition system described in ref. 36. From the model calculations and the reference measurement at the PTB synchrotron beamline at BESSY II, a reflectivity of 14.6% (38.2% per single mirror) under near normal incidence (2 degrees with respect to the normal) at 18.9 nm was determined. The combined reflectivity of all multilayer mirrors (M1-3) is approximately 3.2%.

The experiment was arranged such that incoherent plasma emission, that appears as a more than two orders of magnitude weaker background in the spectrum in Fig. 1c, could potentially only reach the detector via the mirrors aligned for the directed SXRL emission. The distance of 1 m between M2 and the source geometrically selects the directed SXRL emission over the existing 4π plasma emission. Further, the multilayer mirrors spectrally select a narrow portion of the emission centred near the laser line to further reduce possible emission not relating to lasing to reach the target. In the experiment the SXRL emission was optimized regarding the number of pump laser exposures on the same position on the Mo target (see Methods). Subsequent exposures on the same target position generate a microstructure on the initially polished target and the resulting inhomogeneous plasma formed exhibits reduced laser emission. During the experiment, no significant background from this plasma emission in absence of laser emission in case of too many subsequent exposures was observed confirming sufficient selectivity of SXRL emission over plasma emission in the experiment.

For imaging applications and coherence measurements different samples were introduced into the refocused beam. A soft X-ray sensitive CCD captured the diffracted light downstream. The photon flux per shot, measured with a calibrated XUV-sensitive photo diode, is (3.2 ± 0.3) × 10^10^ photons per shot at the source, which leads to an overall capability of the system producing up to 3 × 10^12^ photons per second in less than 0.01% bandwidth centred around 18.9 nm.

## Coherence of the SXRL

**Figure 2:**
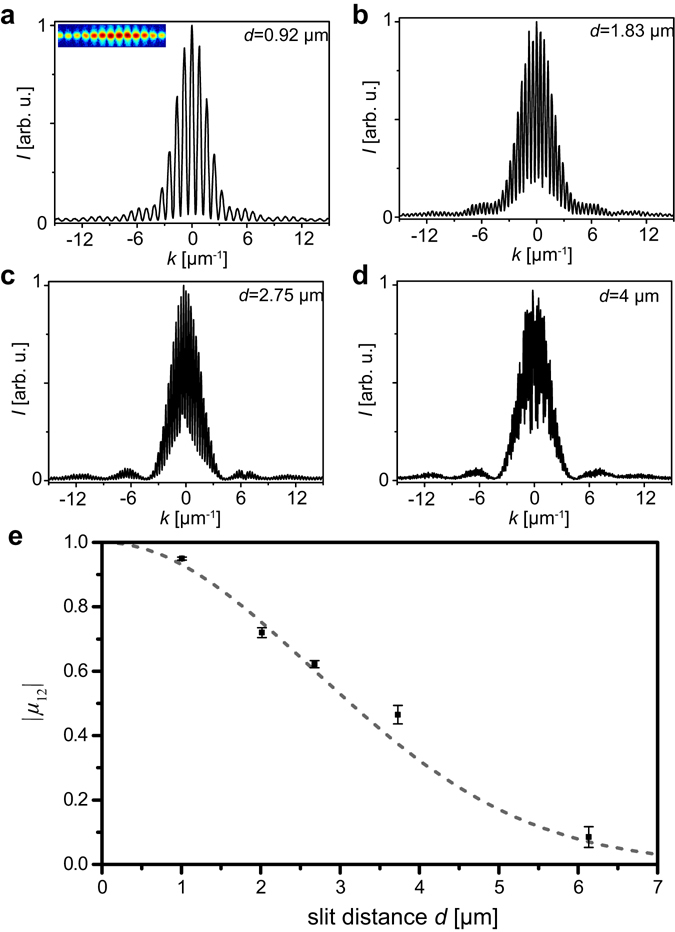
Recorded fringe patterns after a double slit in the refocused beam. Panels (a–d) show characteristic fringe patterns measured for different slit distances *d* as indicated in the panels. The inset in panel (a) shows an example of the raw data captured on the CCD featuring well resolved fringes. (**e**) Modulus of the complex coherence factor *|µ*_12_ *|* for different slit distances along the horizontal axis of the laser. The dotted line shows a Gaussian fit with a width at full width half maximum of 3.13 µm.

For quantification of the transverse coherence of the SXRL radiation 300 nm wide double slits with different slit distances (*d* = 0.92 to 6 µm) were introduced into the refocused beam after the curved mirrors. The fringe patterns were subsequently recorded by a CCD. As a next step the pixels of the CCD were binned down vertically to obtain typical fringe patterns along the horizontal axis of the beam. Typical obtained fringe patterns are depicted in Fig. 2a–d. For increased slit separation, the fringe visibility decreases indicating reduced coherence for wavelets emitted from the slits. As a sanity check that the narrower fringe spacing for larger slit spacing does not reduce the visibility due to possibly limited resolution of the detector, these larger distances were measured at a larger sample to CCD distance, which confirmed the limited visibility.

From scanning diffracting objects across the beam, the beam diameter in the focus was determined to be approximately 15 µm at full width of half maximum (FWHM) resulting in a source size of approximately 30 µm considering that the curved mirrors in the setup demagnify the beam by a factor of two. Hence, homogenous illumination can be assumed for the slit distances discussed here. Fitting the well-known formula, for a two-slit diffraction pattern^37^ at position *z* along the propagation axis

$$I(x)={I}_{0}{[\frac{\sin (\pi \alpha x/\lambda z)}{\pi \alpha x/\lambda z}]}^{2}\{1+|{\mu }_{12}|[\frac{\sin (\pi {\rm{\Delta }}\lambda x/\lambda z)}{\pi {\rm{\Delta }}\lambda x/\lambda z}]\times [\frac{\sin (\pi \delta \beta /\lambda z)}{\pi \delta \beta /\lambda z}]\cos (\frac{2\pi }{\lambda z}\beta x)\},$$

where *α* and *β* are the width and spacing of the slits, respectively, to the experimental data allows deducing the modulus of the complex coherence factor |*μ*_12_|. We assume *I*_0_ to be unity for normalized data and the fringe pattern centered around *x* = 0.

In Fig. 2e the modulus of the complex coherence factor is plotted over the slit distance indicating that the SXRL exhibits partial transverse coherence. By fitting a Gaussian to the experimental data^37^ and use that the transverse coherence diameter *D*_coh_ (diameter of the coherent patch) is defined when |*μ*_12_| = 0.88 (ref. 38) the transverse coherence diameter of the reimaged source can be deduced to *D*_coh_ = 1.3 ± 0.1 μm. Hence, the transverse coherence diameter can be estimated to be in the order of ~8% of the beam diameter. This value agrees very well with a value of 1/20 beam diameters obtained by Liu and co-workers^29^ for a fully saturated nickel-like Cd SXRL as well as the values obtained by Wang and co-workers^28^ for an unseeded high-energy pumped nickel-like Mo SXRL and a neon-like Se SXRL^39^. These numbers further are comparable to the results reported for the FLASH XUV-FEL having a coherence relative transverse coherence diameter of ~6% for multi-shot accumulation^40^ and ~40% for single shot exposure^41^. While the temporal coherence due to the narrow linewidth is outstanding for the presented SXRL source, the transverse coherence limits the usability towards coherent diffraction imaging (CDI). It is important to note that the measurement was performed in the refocused beam, ensuring the highest fluence on the sample and the shortest exposure time for imaging. By doing so, further the Fraunhofer diffraction condition is explicitly satisfied on the double slits, given that in the focus for an ideal beam a flat wave front with infinite curvature can be assumed. This is advantageous for coherence characterization of sources applied for coherent diffraction imaging, because it is known that the transverse coherence of a divergent beam can change over propagation distance^42^. As a result, a far-field measurement of the transverse coherence does not necessarily yield a reliable value for the transverse coherence present in the refocused beam and apart from special cases the transverse coherence is known to be improved in a refocused beam^43^.

**Figure 3:**
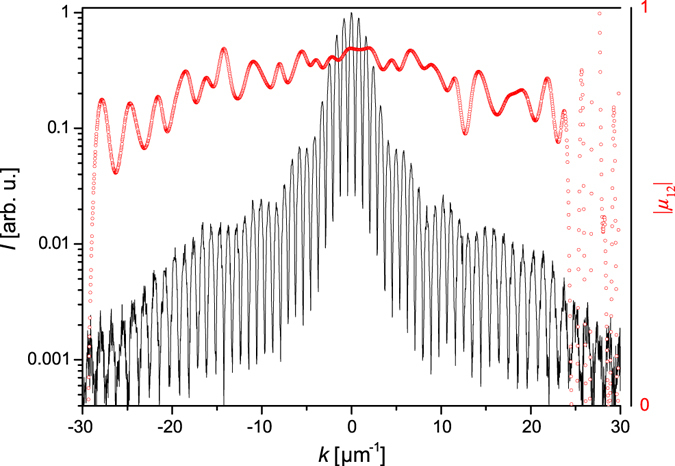
Modulus of the complex coherence factor depending on *k*. The fringes (black solid line) obtained using a slit spacing of *d* = 0.92 µm show excellent contrast up to high momentum transfers. The slight degradation in the mutual coherence factor or fringe visibility (red dots) can be attributed to a limited dynamic range of the detector (~10^3^).

Finally, the temporal coherence or longitudinal coherence length of the SXRL can be estimated. The measured bandwidth of Δ*λ* = 25 pm, as depicted in Fig. 1c, is limited by the resolution of the spectrometer. However, precise measurements of the bandwidth of a Mo-based SXRL using a high resolution spectrometer^25^ yielded a bandwidth of 1.8 pm corresponding to a temporal coherence length of the order of ${l}_{coh}\cong \frac{{\lambda }^{2}}{2{\rm{\Delta }}\lambda }\approx 100\,\mu {\rm{m}}$. Hence, no deterioration of the diffraction pattern due to finite bandwidth of the beam is expected, with the transversal coherence length being substantially smaller. To verify that, a double slit diffraction pattern with the narrowest double slit, having a slit separation of 0.92 µm and a slit width of about 300 nm, was measured in detail. The fringe pattern depicted in Fig. 3 indicates that fringes are observed for very high diffraction angles due to the excellent temporal coherence given that the slit separation is smaller than the transverse coherence diameter. Hence, two wavelets exiting either of the slit observed under large angles, in the presented case 42° with respect to the normal of the sample plane, can still interfere with high fringe contrast. The slight decrease in the mutual coherence factor (red dots in Fig. 3) for high momentum transfers can be attributed to limitations in the dynamic range of the detector (~10^3^).

## CDI using a SXRL

**Figure 4:**
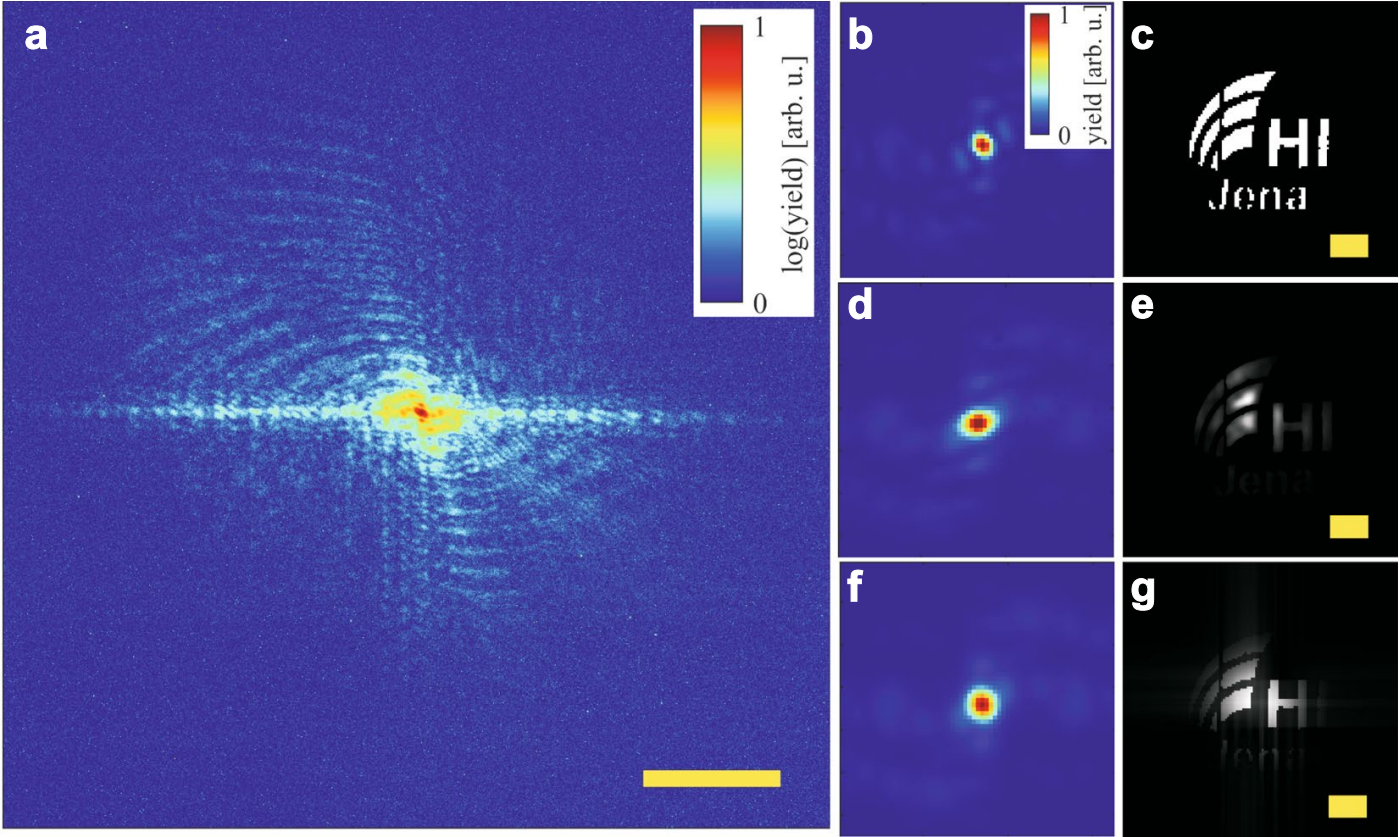
Coherent diffraction imaging using a solid-state SXRL. (**a**) Measured diffraction pattern captured from 300 laser shots featuring fringes extending well to the edge of the detector. (**b**) Comparing the modulus in Fourier space around the central speckle calculated from the STEM image of the sample (**c**) and comparing it to the measured data (**d**) one finds that it is best represented by convoluting the simulated pattern with a Gaussian having a width of 1.7 pixels (**f**) to account for the degree of decoherence. (**g**) The resulting simulation of the object space from the filtered Fourier space compares well to the reconstruction of the experimental data using established phase retrieval algorithms (**e**). The additional amplitude modulation in the retrieved object (Panel (e)) compared to the simulation (**g**) can be explained by the non-uniform wavefront of the SXRL focus (cf. far-field intensity distribution in Fig. 1b). See text for further discussion. Note that panel (a) is plotted using a logarithmic scale, while panels (b,d and f) are plotted on a linear scale. The scale bar in (**a**) is 10 µm^−1^ and those in (**c**), (**e**) and (**g**) are one micron.

As object for coherent diffraction imaging a 50 nm thick silicon nitride membrane with 50 nm gold deposited onto it was employed, where an aperture was fabricated using a focused ion beam (STEM image shown in Fig. 4c). The object was placed in the refocused soft X-ray beam with a large area X-ray-sensitive CCD camera (Andor iKon L, 27.6 × 27.6 mm^2^ chip size) placed 15 mm downstream for capturing the diffracted light. For this geometry, the numerical aperture was 0.67 allowing for a half-pitch resolution of 14 nm. A typical diffraction pattern exhibiting fringes extending to the edge of the detector could be recorded within 300 laser shots (Fig. 4a). Even with a single laser shot diffraction fringes were observed in an area of about 30% of the detector centres around the central speckle.

For the iterative phase retrieval a guided version of the hybrid input-output algorithm was employed that proved to work well in previous CDI experiments^15, 17^. The phase retrieval algorithm was not able to reconstruct the object in full and in the anticipated resolution due to limited transverse coherence of the SXRL (Fig. 4e).

In Fig. 4b to g this is studied in detail by investigating the effect of limited transverse coherence on the central part of the diffraction pattern under the assumption that temporal coherence is not a limiting factor at low spatial frequencies. Comparing the calculated modulus in Fourier space (Fig. 4b) to the measured (Fig. 4d) smearing of the fringes due to limited transverse coherence is observed. For determining the degree of decoherence, the calculated pattern (Fig. 4b) is convoluted with a Gaussian of varying width and the error between calculated and measured pattern is minimized to find best agreement (Fig. 4f). The Gaussian for best agreement was found to have a standard deviation width of 1.7 pixels. A forward calculation of the object space from the diffraction data with limited transverse coherence (Fig. 4g) shows reasonable agreement with the object space reconstructed from experimental data (Fig. 4e). The small discrepancy in the amplitude distribution between Fig. 4e and g potentially arises from a non-uniform illumination amplitude in the refocussed SXRL light. This can be expected from the spatial distribution of the measured far-field profiles (Fig. 1b), which likely translates into the focus. For the simulation (Fig. 4g) a uniform illumination with flat wavefront was assumed.

For the reconstruction presented, the object space support was kept fixed to a support retrieved from the STEM image (Fig. 4c). Hence, a determination of achieved resolution by established methods is not possible. Here it should be stressed that an idealized support is fed into the reconstruction in order to retrieve the coherent contribution of the object space amplitude and observe the limitations due to spatial decoherence. One finds that in the experiment the object can be reconstructed with sufficient amplitude up to a spatial extent of approximately 1.5 microns, which compares well to the result for *D*_coh_ retrieved from a double slit measurement. All distances larger than this in object space result in fringes that cannot be properly phased due to incoherence.

For a direct comparison to a high harmonic source, the same sample was investigated with the same instrument, while the source was switched (details of the laser system and HHG source can be found in ref. 7). The multilayer mirrors were replaced with a pair of mirrors that select the 23^rd^ harmonic at 35 nm wavelength. A diffraction pattern of comparable signal coverage on the CCD as for the SXRL (Fig. 4a) was captured within 600 s or 600.000 laser shots. Since the wavelength was approximately doubled while the geometry was otherwise kept constant, this effectively reduces the achievable resolution by limiting accessible *k* space approximately to a half compared to the SXRL measurement. Due to a power-to-the-four scaling law of integration time versus achievable resolution, this results in 16 times higher required integration time for the HHG source for achieving the same signal in *k* space as with the SXRL, neglecting that in this case the numerical aperture would already be larger than one. Comparing the SXRL, which can run up 100 Hz repetition rate, to a standard kHz-HHG source in terms of achievable resolution with respect to the integration time, we find an advantage of more than three orders of magnitude for the SXRL. For reconstructing the object space from the measured diffraction pattern the same implementation and procedure as described for the SXRL data was used, except for using the shrink-wrap algorithm^44^ to find the support iteratively during the reconstruction without any *a priori* knowledge. See methods section in ref. 17 for further details. The support retrieved by gently applying shrink-wrap constitutes a soft edge of the object allowing an estimate of the achieved resolution (see Supplementary Fig. 1 for a comparison with a fixed support reconstruction). For the SXRL data the fixed support can be considered a soft edge as well, since amplitudes are not retrieved towards the edge of the support which would otherwise mitigate the retrievable resolution.

**Figure 5:**
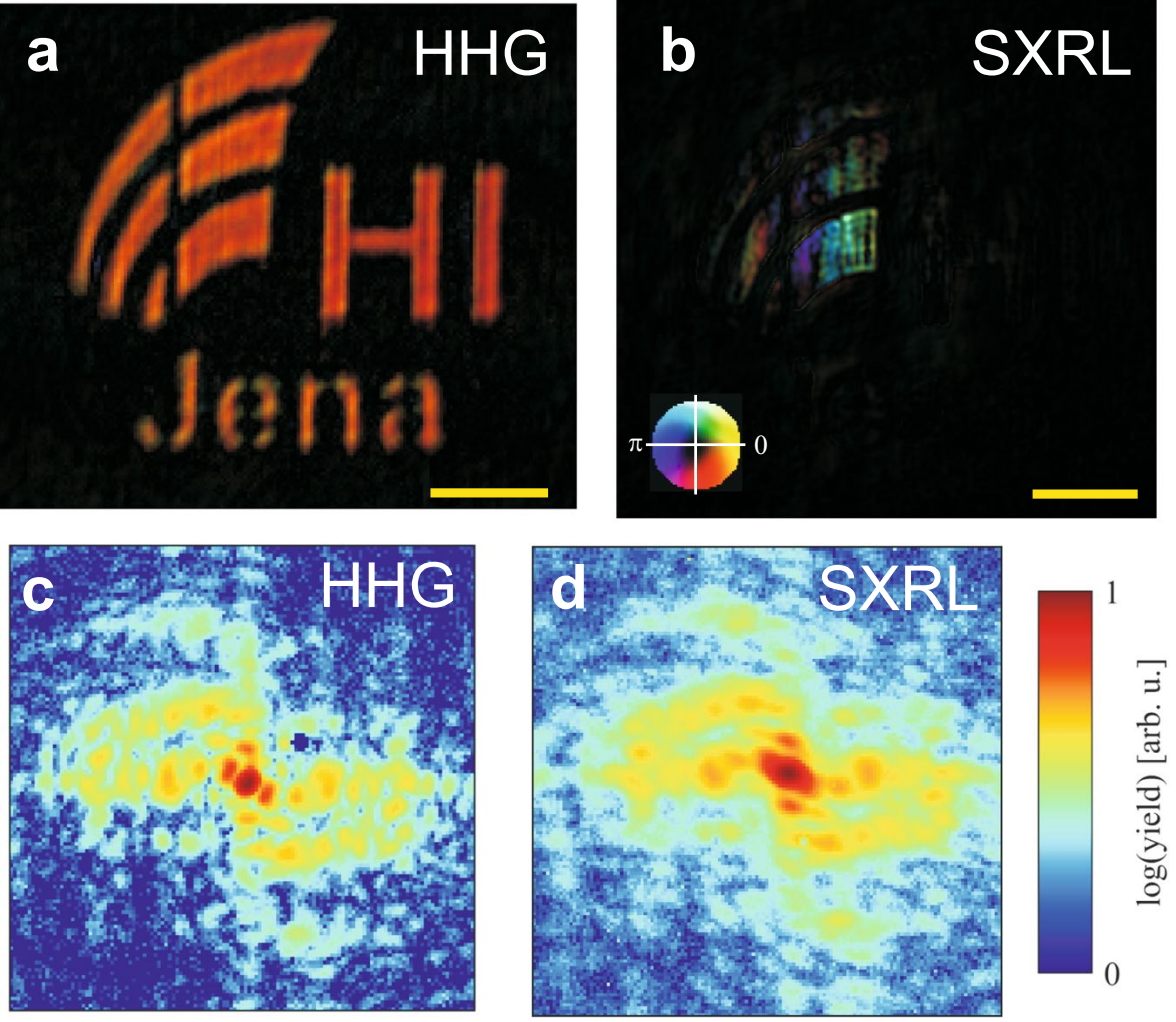
Comparison of CDI using a HHG and SXRL source. The reconstruction of the object from the diffraction pattern captured using a HHG source (**a**) shows the object (Fig. 4c) in detail, while the reconstruction from the SXRL (**b**) is incomplete and features unstable phases. Comparing the raw data measured ((**c**) and (**d**)) the effect of limited transverse coherence of the SXRL becomes obvious. In Panels (a) and (b) the complex-valued object space is depicted where the brightness and hue encode the amplitude and phase respectively (see inset in Panel (b)). The scale bars are one micron. In Panels (c) and (d) the measured intensity of the diffraction pattern around the central speckle is depicted. The image area shown was cropped for easy comparison of the two.

The reconstruction of the HHG experimental data (Fig. 5a) features the object (Fig. 4c) in detail. The smallest features of the sample are well-resolved (width of the vertical sections in letter “n” is approximately 80 nm wide). In contrast, the reconstruction of the SXRL measurement (Fig. 5b) features only an approximately 1.5-micron large fraction of the object as discussed before. Because the phase is not stable across the object the support was kept fixed in the reconstruction of the SXRL data. This could hint at significant fluctuations of the SXRL wavefront as would be expected from the complex shape of the far-field beam profile of the SXRL (Fig. 1b). Directly comparing the diffraction patterns (Fig. 5c and d) shows the effects of limited transverse coherence. From the direct comparison of the CDI reconstruction and the quantitative measurement of the transverse coherence, it can be concluded that for plane wave CDI applications the modulus of the complex coherence factor needs to be larger than 0.75 for a given object size. It is worth noting that the measured limiting value for the complex coherence factor constitutes a lower bound required. For more realistic samples exhibiting hard-edges but non-zero background such as for instance binary masks and lithographic structures^8, 45^ in reflection geometry CDI but also for soft-matter samples exhibiting soft edges this estimate remains valid (see also Supplementary Fig. 2). However, in general a higher photon flux or a better signal-to-noise ratio on the detector will be required for achieving comparable imaging conditions due the lower scattering cross section of such samples.

**Figure 6:**
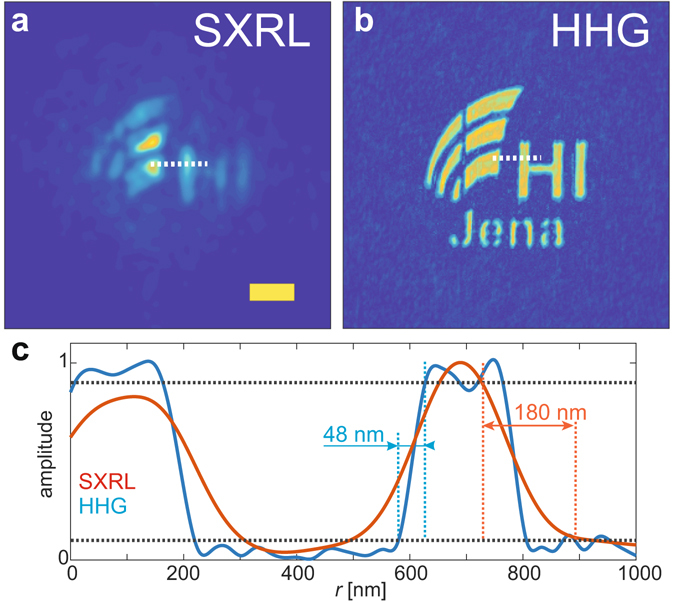
Comparison of imaging resolution. Panels (a) and (b) show the modulus of the reconstructed object space for the SXRL and HHG measurement, respectively. The scale bar in Panel (a) is one micron for both panels. (**c**) The achieved resolution for HHG-CDI is 48 nm (blue line) and approximately 180 nm for the SXRL-CDI measurement (red line). The line profiles were taken at the position of the dotted white line in Panels (a) and (b), respectively.

An important measure for the quality of a CDI measurement is the achieved transverse resolution. A comparison for the CDI data taken with a SXRL and HHG source are shown in Fig. 6a and b, respectively. The advantage of the HHG source is evident, while the limited transverse coherence for the SXRL source allows only a glimpse on the shape of the object. For a quantitative analysis line profiles were taken around the left vertical bar of the letter “H” (Fig. 6a and b, indicated by white dotted line), which is measured by STEM to be 200 nm wide, and analysed at 90/10% level^46^ (Fig. 6c). For the HHG source a resolution of 48 nm was determined, while the SXRL reconstruction features approximately 180 nm spatial resolution. Due to the high degree of transverse incoherence, the resolution in the SXRL case (Fig. 6a) is not constant over the object and using alternative methods such as measuring the cut-off of the phase retrieval transfer function is prohibited here due to using a fixed support constraint. Hence, for this particular measurement using the SXRL only a rough estimate for the achieved transverse resolution can be made. It is important to note that for both experiments the speckles in the diffraction patterns captured extended well beyond a numerical aperture of 0.5. Assuming a fully coherent signal one would expect a resolution in the order of the wavelength. In the HHG experiment the achieved resolution amounts to 1.4 wavelengths. The limitation here is likely related to the temporal coherence of the source, i.e. the linewidth of the harmonic line (Δ*λ*/*λ* ≈ 1/30), that effectively limits the resolution^17^. For the SXRL the resolution expected from the extent of the fringes (Fig. 4a) and the wavelength is at least 20 nm, which is about an order of magnitude away from the value achieved in the reconstruction. We attribute this to the limited transverse coherence of the SXRL source. The convergence and quality of the reconstruction is severely limited by the fact that the object is much larger than the coherence length of the source. It is expected that for an object smaller than the transverse coherence diameter under otherwise identical illumination conditions the object can be readily reconstructed at a resolution near the value expected from the largest measured momentum transfer *k*. This is further indicated by the excellent fringe modulation measured for a double slit spacing of 0.92 µm (Fig. 2a).

Established methods in iterative phase retrieval, such as shrink-wrap, that allow retrieval of the object without *a priori* information are thus prone to fail in this case a sharp edge of the object cannot be determined. This constraint can be resolved if the SXRL is used in conjunction with ptychography^47, 48^ or if isolated objects are imaged that are smaller than 1.5 micron, which is also suggested by high fringe contrast obtained from the SXRL for sufficiently small slit spacing (Fig. 2a).

## Summary

In summary, we have studied the transverse coherence properties of a transient nickel-like Mo soft X-ray laser pumped at moderate energies and applied this source for coherent diffraction imaging. A direct comparison to a high harmonic source indicates that the extraordinary high flux of the SXRL allows for a more than three orders of magnitude shorter integration time for collecting diffraction data that can potentially result in the same imaging resolution. A drawback of using a SXRL for table-top CDI is the fixed wavelength and the limited transverse coherence diameter rendering only a fraction of the wavefront useful for CDI. Direct comparison to a double slit experiment suggests that the modulus of the complex coherence factor of |*µ*_12_| > 0.75 is required for plane wave CDI. For measuring the coherence properties, it is beneficial to measure the coherence directly in the refocused beam, i.e. the reimaged source, as this omits propagation effects of the coherence properties. In this case a known complex-shaped aperture and established algorithms for phase retrieval in coherent diffraction imaging can be employed to estimate the transverse coherence properties given that the diffracting object is larger than *D*_coh_. For the experiment presented the transverse coherence limits the object size or possible field of view for CDI to 1.5 microns.

The presented approach of employing a SXRL pumped at moderate energies enables scaling the concept to much higher repetition rates (up to 1 kHz, cp. ref. 49). Under the conditions of pumping the SXRL at moderate laser energies (<500 mJ) comparable coherence properties as SXRLs pumped with energies exceeding 1 J is confirmed. Further it is shown that the transverse coherence of the SXRL compares to those of SASE-FELs for multi-shot exposure. Future directions in using SXRLs for single-shot and high resolution diffraction imaging might employ seeding with a high harmonic source^28, 50, 51^, which might combine the best of two worlds. Despite the observed limitations the presented source and scheme offers a wealth of possible applications, e.g., in imaging of quantum dots or lithographic mask inspection in combination with ptychographic scanning technique.

## Methods

### Molybdenum soft X-ray laser

The soft X-ray laser (SXRL) operating in grazing incidence pump (GRIP) geometry was pumped by two pulses of a high repetition rate 100 Hz thin disk laser (TDL) chirped pulse amplification (CPA) system. The TDL system consists of a front-end with an Yb:KGW oscillator, stretcher and Yb:KGW regenerative amplifier followed by two regenerative amplifiers and one multipass amplifier. The frontend delivers an output energy of 0.3 mJ, at a pulse duration of about 1.5 ns at 1030 nm. The output is divided into two pulses and subsequently each of these is amplified in a regenerative amplifier to a level of about 100 mJ. The pulse from the first regenerative amplifier is compressed to a duration of approximately 150 ps using a grating compressor, the output of the second regenerative amplifier is fed into a thin disk multipass amplifier which amplifies the pulses to an energy up to 400 mJ and which is subsequently compressed in a grating compressor to about 2 ps pulse duration. The long pre-pulse (150 ps, *E* ≈ 70 mJ) is focused by a cylindrical (*f* = −500 mm) and a spherical lens (*f* = 380 mm) onto the target at normal incidence giving a line focus of about 30 µm in width. The generated plasma column will then be heated by a short pulse (2 ps, *E* ≈ 270 mJ) focused according to the GRIP method by a spherical mirror (Edmund Optics, *f* = 762 mm) into the preformed plasma. For the Mo target an optimum GRIP angle of 24 degree was determined. The delay between the two pulses has appeared as a very critical parameter^21, 22, 52^. Therefore, the delay between long and short pulse can be adjusted by adapting the round-trip time of the two regenerative amplifiers as well as fine tuning by an additional delay stage. Because of the high repetition rate all optical components are protected against debris by thin glass plates or foils, and the SXRL output is guided through an aperture to reduce debris contamination on the following optical elements. A Mo slab target with a length of 50 mm and a width 5 mm is used in the experiments. The target was attached to a motorized stage with four degrees of freedom allowing the adjustment in three axes as well as the continuous renewing of the target surface by translating the slab. The most stable SXRL operation was found if the target surface was renewed after 5–10 laser shots. The spectral output of the SXRL has been measured using a flat field grating spectrometer. It consists of a filter wheel equipped with Al filters (thickness 0.2 to 1 µm), an entrance slit (100 µm), an aberration corrected concave grating (HITACHI #0437, 1200 l/mm) on a rotational stage and a back-illuminated CCD camera (ANDOR, DO420A-BN,1024 × 256 pixel). For a 70 mJ/150 ps long pulse and 270 mJ/2 ps short pulse a lower limit for the energy of one single SXRL pulse was estimated to 300 nJ and a divergence of about 10 mrad.

## Electronic supplementary material

Supplementary Information
