# Supplementary material for: Transverse Coherence Limited Coherent Diffraction Imaging using a Molybdenum Soft X-ray Laser Pumped at Moderate Pump Energies
Source: Sci Rep. 2017 Jul 13;7:5314. doi: 10.1038/s41598-017-05789-w (PMC5509821; doi:10.1038/s41598-017-05789-w)
Supplement: Supplementary file 1 — Supplementary Information [file 41598_2017_5789_MOESM1_ESM.pdf]

Supplementary Information

Transverse Coherence Limited Coherent Diffraction Imaging using a Molybdenum Soft X-ray Laser Pumped at Moderate Pump Energies

M. Zürch[1,2,3,*], R. Jung[4], C. Späth[5,6], J. Tümmler[4], A. Guggenmos[5,6], D. Attwood[7], U. Kleineberg[5,6], H. Stiel[4] and C. Spielmann[1,3,*]

[1]Institute of Optics and Quantum Electronics, Abbe Center of Photonics, Friedrich Schiller University Jena, Max-Wien-Platz 1, 07743 Jena, Germany

[2]University of California Berkeley, Chemistry Department, Berkeley, CA 94720, USA

[3]Helmholtz Institute Jena, Fröbelstieg 3, 07743 Jena, Germany

[4]Max-Born Institute, Max-Born Str. 2A, D-12489 Berlin, Germany

[5]Ludwig-Maximilians-Universität München, Am Coulombwall 1, D-85748 Garching, Germany

[6]Max-Planck-Institut für Quantenoptik, Hans-Kopfermann-Str. 1, D-85748 Garching, Germany

[7]University of California Berkeley, Department of Electrical Engineering and Computer Sciences, Berkeley, CA 94720, USA

[*] Correspondence should be addressed to: mwz@berkeley.edu (M. Z.); christian.spielmann@uni-jena.de (C. S.)

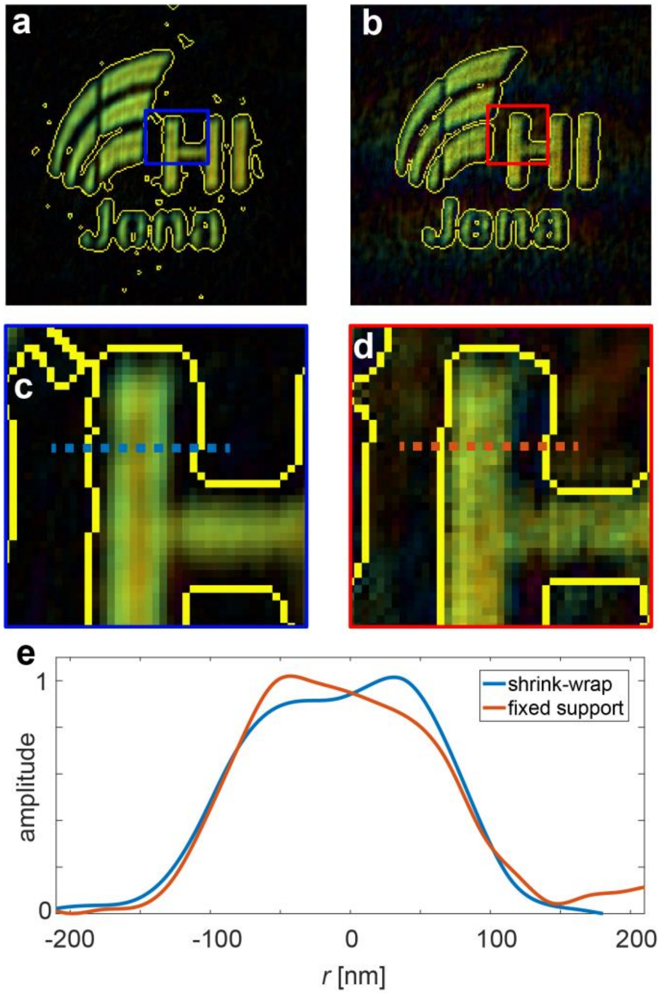

Supplementary Fig. 1. Reconstruction of HHG-CDI data with dynamic support (shrink-wrap) and fixed support. (a) Reconstruction of HHG-CDI data using a dynamic support constraint, i.e. shrink-wrap. The final support is indicated by the thin yellow line that is overlaid with the retrieved object space. (b) Same as (a) but with the same fixed support constraint as used for retrieving the SXRL-CDI data. Panels (c) and (d) are magnified regions of panels (a) and (b), respectively, indicated by the blue and red squares. The dynamically retrieved support is very similar to the one derived from the STEM image. The small differences likely account for deviations of scattering/absorption cross sections at the edges between XUV radiation and electrons. Thus, the dynamic support matches more precisely the actual diffraction data leading to an overall less noisy impression of the reconstructed object space. (e) Taking line profiles (positions indicated by dotted lines in panels (c) and (d)) shows that the resolution for dynamic retrieved support (blue line) and for fixed support (red line) is comparable and hence does not limit the comparability to the SXRL-CDI experiment, where only a fixed support constraint allowed estimating the object. In panels (a)-(d) the brightness and hue encode the amplitude and phase of the retrieved complex object space (cf. also main text Fig. 5). A constant phase was added in the reconstruction with dynamic support to align the hue for better comparison.

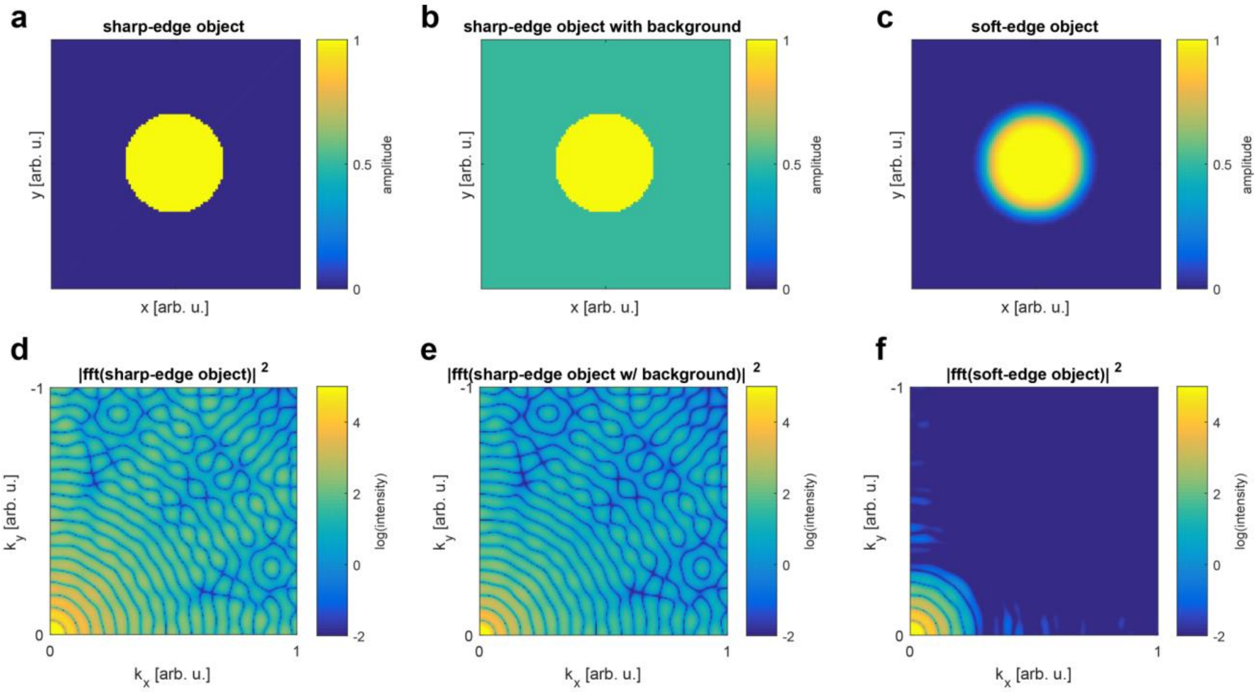

Supplementary Fig. 2. Simulation for different object edges and backgrounds. Panels (a)-(c) depict circular test objects in object space for which fully coherent plane wave illumination is assumed. Panel (a) depicts a circular object with sharp edges. The object in Panel (b) features sharp edges and constant background emulating a sharp-edged object on a partially transparent substrate. It also equals a sharp-edged highly reflecting object on a flat lower reflecting substrate in reflection geometry. Panel (c) instead is the same object as in Panel (a), but is soft-edged by a Gaussian filter emulating a soft-edge object such as a cell. Panels (d)-(f) show a quadrant of diffraction intensities calculated for the objects corresponding to the object depicted above. As can be seen, for the sharp-edge object (Panels (a) & (d)) and the sharp-edge object with background (Panels (b) & (e)) the fringe contrast and fringe scaling for increasing k (k=0 is the lower left corner of each panel in the bottom row) are identical and only the overall yield is slightly reduced. In contrast, for the soft-edge object (Panel (c) & (f)), that is otherwise identical to the sharp-edge object, one finds that the fringe contrast is preserved as well (for better comparison the color scales are kept identical). However, the radial intensity scaling of the fringes from k=0 is reduced significantly. This indicates that for capturing soft-edged objects one requires significantly more photon flux and/or better signal-to-noise for detection in order to capture the weak fringes at higher k due to the lower scattering cross section of soft edges. The plotted scale is logarithmic in panels (d)-(f) and linear in panels (a)-(c).
